# Detection of Personal and Family History of Suicidal Thoughts and Behaviors using Deep Learning and Natural Language Processing: A Multi-Site Study

**DOI:** 10.21203/rs.3.rs-4014472/v1

**Published:** 2024-03-11

**Authors:** Prakash Adekkanattu, Al’ona Furmanchuk, Yonghui Wu, Aman Pathak, Braja Gopal Patra, Sarah Bost, Destinee Morrow, Grace Hsin-Min Wang, Yuyang Yang, Noah James Forrest, Yuan Luo, Theresa L. Walunas, Wei-Hsuan Lo-Ciganic Jenny, Walid Gelad, Jiang Bian, Yuhua Bao, Mark Weiner, David Oslin, Jyotishman Pathak

**Affiliations:** 1 Weill Cornell Medicine, New York, NY, USA; 2 Northwestern University Feinberg School of Medicine, Chicago, IL, USA; 3 University of Florida College of Medicine, Gainesville, FL, USA; 4 Lawrence Berkeley National Laboratory, Berkeley, CA, USA; 5 University of Pittsburgh School of Medicine, Pittsburgh, PA, USA; 6 Corporal Michael J Crescenz Veterans Affairs Medical Center, Philadelphia, PA, USA

## Abstract

**Objective:**

Personal and family history of suicidal thoughts and behaviors (PSH and FSH, respectively) are significant risk factors associated with future suicide events. These are often captured in narrative clinical notes in electronic health records (EHRs). Collaboratively, Weill Cornell Medicine (WCM), Northwestern Medicine (NM), and the University of Florida (UF) developed and validated deep learning (DL)-based natural language processing (NLP) tools to detect PSH and FSH from such notes. The tool’s performance was further benchmarked against a method relying exclusively on ICD-9/10 diagnosis codes.

**Materials and Methods:**

We developed DL-based NLP tools utilizing pre-trained transformer models Bio_ClinicalBERT and GatorTron, and compared them with expert-informed, rule-based methods. The tools were initially developed and validated using manually annotated clinical notes at WCM. Their portability and performance were further evaluated using clinical notes at NM and UF.

**Results:**

The DL tools outperformed the rule-based NLP tool in identifying PSH and FHS. For detecting PSH, the rule-based system obtained an F1-score of 0.75 ± 0.07, while the Bio_ClinicalBERT and GatorTron DL tools scored 0.83 ± 0.09 and 0.84 ± 0.07, respectively. For detecting FSH, the rule-based NLP tool’s F1-score was 0.69 ± 0.11, compared to 0.89 ± 0.10 for Bio_ClinicalBERT and 0.92 ± 0.07 for GatorTron. For the gold standard corpora across the three sites, only 2.2% (WCM), 9.3% (NM), and 7.8% (UF) of patients reported to have an ICD-9/10 diagnosis code for suicidal thoughts and behaviors prior to the clinical notes report date. The best performing GatorTron DL tool identified 93.0% (WCM), 80.4% (NM), and 89.0% (UF) of patients with documented PSH, and 85.0%(WCM), 89.5%(NM), and 100%(UF) of patients with documented FSH in their notes.

**Discussion:**

While PSH and FSH are significant risk factors for future suicide events, little effort has been made previously to identify individuals with these history. To address this, we developed a transformer based DL method and compared with conventional rule-based NLP approach. The varying effectiveness of the rule-based tools across sites suggests a need for improvement in its dictionary-based approach. In contrast, the performances of the DL tools were higher and comparable across sites. Furthermore, DL tools were fine-tuned using only small number of annotated notes at each site, underscores its greater adaptability to local documentation practices and lexical variations.

**Conclusion:**

Variations in local documentation practices across health care systems pose challenges to rule-based NLP tools. In contrast, the developed DL tools can effectively extract PSH and FSH information from unstructured clinical notes. These tools will provide clinicians with crucial information for assessing and treating patients at elevated risk for suicide who are rarely been diagnosed.

## 1. Introduction

Suicide represents a critical public health challenge globally, ranking as the fourth leading cause of death among people between 15 and 29 years old^1^. In the United States, it is the tenth leading cause of death overall and ranked second for those aged 10 to 34, as reported by the Centers for Disease Control and Prevention^2^. In 2020, suicide rate among U.S. veterans – a high-risk population – was 57.3% higher than in the non-veteran adult population, when adjusted for age and sex differences^3^. However, patients at risk of suicide are often underdiagnosed for a variety of reasons such as stigma associated with mental health issues and the limitations of diagnostic codes in capturing the complexity of mental health conditions. Recognizing warning signs and addressing risk factors with effective early interventions are crucial in suicide prevention and mental health promotion^4,5,6,7^. Personal suicide history and a family suicide history (PSH and FSH, respectively) significantly increase future suicide risk, emphasizing the importance of identifying these factors^8,9^.

Based on the existing literature, suicidal thoughts and behaviors (STBs) broadly encompasses thoughts, behaviors, actions, and emotions linked to suicide and self-harm^10^. STB manifests in various forms, including suicidal ideation (SI), suicidal behavior (SB), and suicide attempts (SAs), each carrying distinct implications for individuals. SI entails a range of thoughts and preoccupations with death and suicide, varying in intensity from fleeting thoughts to detailed plans^11^. Although its definition may vary and often evolves, SB generally refers to any self-directed actions with potential lethality, ranging from preparatory acts to completed suicides^10^. Thus, it is critical to develop effective methods to identify individuals with STB that often are underdiagnosed to improve the management of suicide prevention and treatment.

Electronic health records (EHRs) have been widely used to study phenotyping and risk prediction models^12^. Notably, the majority of suicide decedents had a physician visit in the year prior to their death and 45% had a visit in the last month of life, highlighting the potential of EHR data in suicide risk prediction and prevention^13^. While International Classification of Disease (ICD-9/10) diagnosis codes in EHRs are used to document STB, their sole use has proven insufficient^14^. This inadequacy is partly because details like personal and family history of STB are often only recorded in clinical notes instead of ICD-9/10 diagnoses codes. The ICD-9 had no defined diagnosis code to document the historical aspect of STB. Personal history of self-harm was introduced only in 2016 when ICD-10 was formally adopted by EHR systems. Natural language processing (NLP), machine learning (ML), and deep learning (DL) techniques show promise in effectively mining clinical notes for clinical information that are not readily available in structured data. Most recently, transformer-based architectures have facilitated building high performance models and fine-tuning has made it possible to effectively utilize these models for a wide variety of tasks. Since these language models (e.g., BERT, ChatGPT, LLaMA, FLAN) have been trained using very large datasets, they possess contextual knowledge, and fine-tuning them with problem-specific data can achieve substantial improvement in performance^15^. In particular, the BERT (Bidirectional Encoder Representations from Transformers) models effectively capture the interaction between a key clinical concept and their surrounding context, and have shown superior performance in various NLP tasks including text classification^16^.

Advancements in automated detection of SI, SB, and SA from clinical notes have shown varied success^17,18,19,20,21,22,23,24,25,26^. These approaches, ranging from rule-based algorithms to ML techniques, primarily focus on identifying suicidal tendencies without considering their temporal aspects, often conflating current and historical events. Notably, there has been a lack of emphasis on distinguishing personal and family histories of STB, despite the critical but different insights each of these factors offer. Our study addresses this gap by developing and evaluating novel NLP and DL-based tools. These tools are designed to detect both PSH and FSH from clinical narratives, tested across diverse patient cohorts in three academic medical centers in the U.S.

## 2. Materials and Methods

### 2.1 Study Setting and Data Sources

The rule-based NLP and DL tools were developed and validated at three academic medical centers: Weill Cornell Medicine (WCM), Northwestern Medicine (NM), and the University of Florida Health (UF) to enhance the generalizability and portability of our tool. This multi-site study was conducted with the approval of the Institutional Review Boards (IRBs) at each participating site and by a central IRB, ensuring adherence to ethical standards and patient privacy regulations. In order to enhance the generalizability of the NLP and DL tools, we assembled clinical notes from diverse patient cohorts seeking care from outpatient ambulatory services, emergency department (ED), inpatient care from multiple specialties. In the gold standard corpora from each site, we gathered demographic data and recorded diagnoses of SI and SB for the respective patients.

The training data for this study was sourced from WCM, an academic medical center in New York City affiliated with NewYork-Presbyterian Hospital. The dataset comprised of more than 13.8 million clinical encounter notes derived from patients (N=177,993) who were either prescribed antidepressants or diagnosed with mental health conditions between 2000–2020. Clinical notes consisted of progress notes (49.4%), telephone encounters (32.3%), patient instructions (2.1%), letters (2.0%), nursing notes (0.4%), and unknown types (13.8%). The notes, authored by clinicians from various specialties including internal medicine, psychiatry, anesthesiology, and pain medicine, offer a rich, unstructured collection of information, reflecting the diversity of clinical environments and the variability levels of detail provided.

NM is a comprehensive academic medical center located in Chicago, IL. The NM Enterprise Data Warehouse is an integrated data platform that provides secure, centralized access to clinical and ancillary data sources from all inpatient and outpatient settings. It consolidates data from Northwestern Memorial HealthCare, the Feinberg School of Medicine at Northwestern University, and Northwestern Medicine Regional Medical Group. The 400 notes used for validation were randomly collected from the integrated system between January-December, 2018. The gold standard corpus consisted of 3 (1%) assessment & plan notes, 23 (7.3%) consult notes, 4 (1.3%) discharge/summary notes, 21 (6.7%) ED notes, 26 (8.3%) History & Physical notes, 12 (3.8%) plan of care notes, 167 (53.4%) progress notes, 63 (20.1%) psychiatric note, 3 (1%) telephone encounters, and 16 (5.1%) notes of other types. The clinical notes used for validation were written by 66 (21.1%) psychiatric specialists and 247 (78.9%) non-psychiatric specialists.

The UF Health Institutional Data Repository (IDR) is a clinical data warehouse that aggregates data from the university’s various clinical and administrative information systems, including the Epic (Epic Systems Corporation) system. At UF, the corpus was developed using 400 clinical notes from a cohort of individuals with at least one prescription of opioids between 2016 and 2019 recorded in the IDR. Patients with pain conditions or those prescribed opioids are at an increased risk of STBs. Utilizing a patient sample with unique characteristics can improve the model’s generalizability. Adult patients aged ≥18 who had at least one outpatient visit and at least one eligible opioid prescribing order (excluding injectable and buprenorphine approved for opioid use disorder) were included in the patient sample. The gold standard corpus consisted of 13(3.3%) consult notes, 6(1.5%) discharge summary, 11(2.8%) ED notes, 10(2.5%) History & Physical notes, 319(79.8%) progress notes, 8(2.1%) psychiatric inpatient notes, and 33(8.4%) other or unknown types.

### 2.2 Evaluation of the NLP and DL tools

The performance of the rule-based NLP and DL tools was evaluated using a gold standard corpus developed by manual annotation. We used the manual in-file annotation (NM) and Brat annotation tool (WCM, UF) to identify relevant concepts in notes collected from the corresponding EHR systems^37^. At each site, we set up a secure instance of the annotation tool with the same annotation scheme. All annotators were given the same annotation guidelines. These guidelines (Exhibit E1, Supplementary Materials) include finding all mentions of history of STB and family history of STB within the note and then classifying the note based on a majority polling of various instances and whether they are affirmative or negated. When there is only one instance of “history of STB” or “family history of STB” annotated in a given note, we classify the note based on whether that instance is negated or not. However, when there are multiple instances in a given note, we use a majority poling among those instances. When there is a tie between positive and negative instances, we classify the note based on the last-mentioned instance. After both annotators completed annotations on the documents, any disagreements between the two sets were resolved through joint sessions of annotations, giving us a final gold standard annotated document set.

At WCM, we developed the gold standard through a manual review of 301 encounter notes selected at random from a superset of notes containing the character string “suicide”. These notes were not part of the 1000 notes previously selected for the development and testing (§2.3). To establish the reference standard, two reviewers (PA, BGP) annotated all notes based on the previously defined guidelines. In instances of differing assessments, reviewers resolved discrepancies through joint sessions. The reviewers also confirmed all notes that did not have any mention of suicide history information. At NM, manual annotation of 400 notes was performed by three reviewers (YY, NF, AF). Four labels were created to identify the presence or absence of a personal and family history of STB at each note. Each note was annotated independently by two reviewers. If the two reviewers differed in their assessment (Table S2, Supplementary Materials) the discrepancy was resolved through a joint session of all three reviewers. At UF, a total of 400 notes were identified for manual annotations. Three annotators (AP, ML, SZ) classified each note for the presence or absence of STB. Discrepancies among the annotators were resolved through discussions to reach a consensus.

During the validation of annotation, we observed generally a high inter-rater agreement between the manual annotators at all three sites. At WCM, the Cohen’s Kappa measured was 0.89 for PSH and 0.89 for FSH. Before the final voting process among all three reviewers at NM, the Cohen’s Kappa coefficient were 0.75 and 0.85 for PSH and FSH, respectively. At UF, the Cohen’s Kappa score of 0.89 was observed for PSH and FSH combined, ensuring sufficient agreement between reviewers.

### 2.3 Rule-based NLP tool development

To develop a novel rule-based tool, we employed the Leo NLP framework, an open-source tool provided by the U.S. Veterans Administration^27^. Specifically, we created a dedicated instance of Leo, termed *SuicideHistoryExtractor*, to detect historical instances of STB from clinical notes. Our approach involved two distinct pipelines to extract PSH and FSH, utilizing a dual lexicon strategy. This strategy comprised target STB and key historical modifiers to accurately identify and contextualize suicidality references (Table S3, Supplementary Materials).

**Figure 1.**
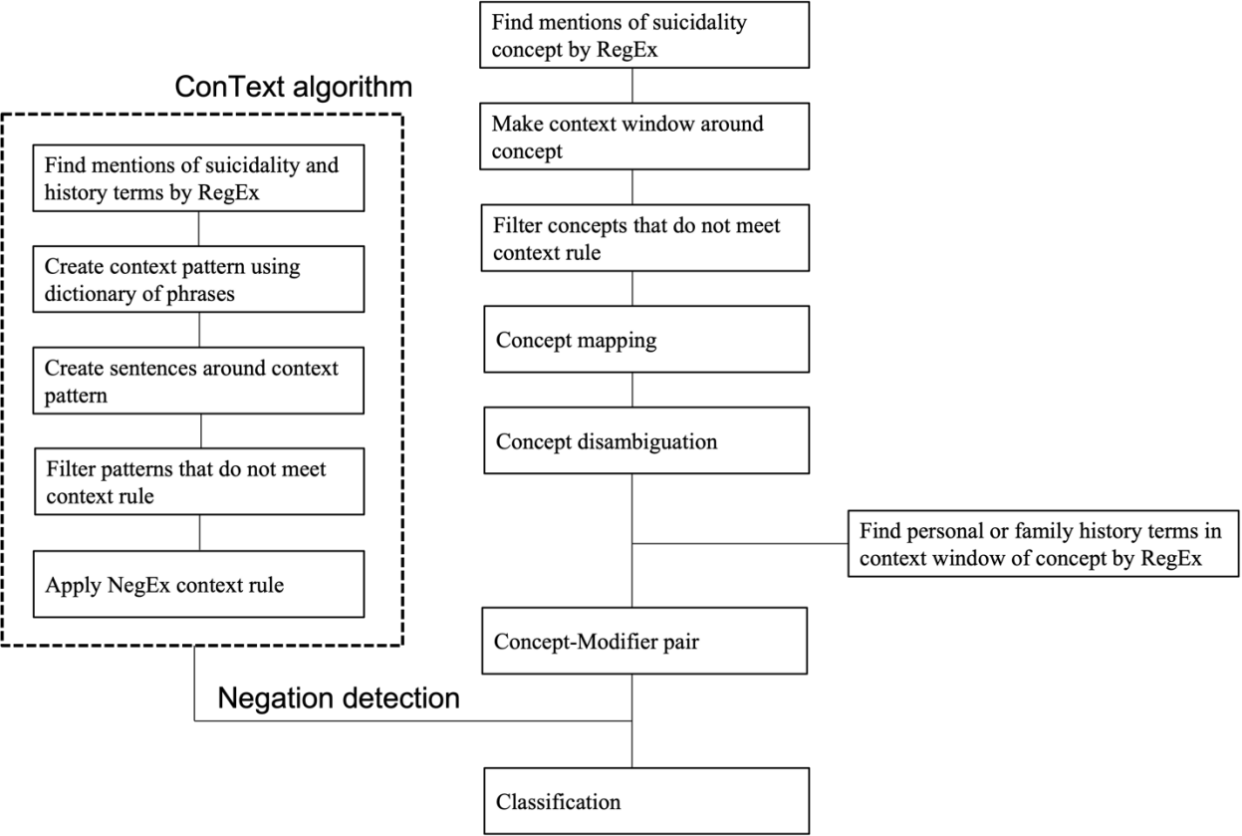
Rule-based NLP algorithm implemented for detection suicide history in clinical notes

We developed an extraction logic for identifying STB concepts through a structured, iterative process. Initially, we focused on defining key terms associated with STB, including SI, SB, and SA while excluding non-life-threatening behaviors like cutting and burning. Utilizing string matching, filters, and a series of regular expressions, we captured various expressions of these concepts (Table S3, Supplementary Materials). Context analyses were then performed by concept mapping, disambiguation, and filtering. We then identified historical indicators of STB using modifiers and paired these with core concepts using regular expressions and string matching. The ConText algorithm was employed to discern negated instances^28^. For FSH, we searched for family-related terms within a defined proximity of the concept-modifier pair (Table S3, Supplementary Materials). The final document classification combined majority polling and analysis of the last mentioned in the document. This comprehensive approach, illustrated in Figure 1, ensured accurate and context-sensitive extraction of STBs.

For the rule-based algorithm development, we selected a sample of 1,000 notes, randomly chosen from a larger set previously flagged for containing the string “suicide”, from WCM EHR. This selection strategy was aimed at enriching our dataset with more instances of suicide history, a relatively rare occurrence in general clinical documentation. The sample was divided into four batches of 100, 200, 300, and 400 notes for detailed analysis. Through this process, we continuously identified and addressed shortcomings in our algorithm, particularly in areas of lexicon usage, context analysis, and rule-based filtering and validation. This iterative refinement was conducted until the algorithm consistently and accurately extracted all relevant instances of history of STB.

For each note, the NLP method attempted to extract all instances of history of STB. For notes with multiple instances, we applied a majority polling heuristic for document-level classification, consistent with the approach used in creating our gold standard (§2.2). In cases where a single instance of “STB history” or “family STB history” was identified, classification was based on the presence or absence of negation. For notes with multiple instances, the majority and last-instance rule was applied. This methodology was uniformly applied across both PSH and FSH data extraction processes.

In each participating institute, the *SuicideHistoryExtractor* was deployed to analyze notes from the corpuses defined above (§2.1), and the system-level performance was evaluated using the gold standard corpus detailed above (§2.2). The NLP system produced two types of outputs: a raw output enumerating extracted entities, and a classification output providing a document-level classification for each note. The effectiveness of the rule-based tool was evaluated by comparing its classifications against the gold standard, using precision, recall, and F1-score metrics.

### 2.4 Transformer based DL tool development

**Figure 2.**
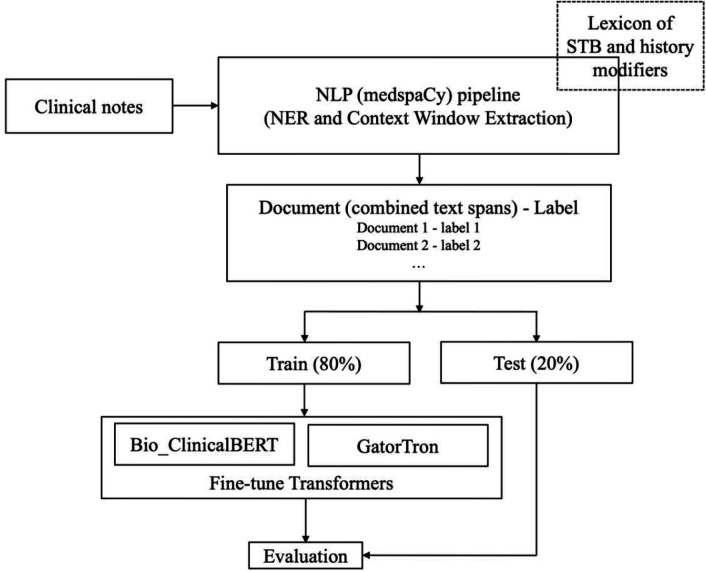
Architecture of the transformer - based method implemented for detecting PSH and FSH in clinical notes.

We employed the BERT framework to construct a transformer-based classifier for analyzing history of STB in clinical texts, as illustrated in Figure 2. The classifiers, pre-trained on extensive data in clinical domains, are adept at understanding context, and their effectiveness is further enhanced by fine-tuning to capture intricate interactions between clinical concepts and their contextual environment. We used a custom NLP pipeline to transform the raw text into a smaller string with key concepts and their surrounding words. The raw text instances are first tokenized using a clinical domain tokenizer implemented in the medspaCy library^29^. Key concept (anchor n-gram) for history of STB is identified using NER TargetRule in medspaCy. The target rule was developed using a dictionary of terms and phrases based on the concept-modifier identified in our rule-based NLP algorithm for PSH and FSH. A context window of *n* words to the left and *n* words to the right of the matched concept are then extracted. A given document may have multiple instances of concept terms and we extracted text spans for all those instances. These text spans were then combined to form a text representation of that document and used for further modeling. Such an approach generally works well for clinical documents where majority of text describe other aspects of patient care and may not be directly relevant to the specific concept of interest. We assigned labels to the combined text same as the label assigned by the annotators for the whole document. The optimum context window size was determined by *n* varying from 8, 16, 24, and 32 words to find the best performance. For our classification task, we used the Bio_ClinicalBERT^30^ and the GatorTron^31^ models; both of which had shown good performance when applied to clinical notes. We used Hugging Face’s transformers library^32^ to initialize both these models and fine-tune them using code written in PyTorch^33^. The Bio_ClinicalBERT was pretrained on MIMIC-III^34^ and the GatorTron model was pretrained on EHR notes at UF^31^. We trained both models using a batch size of 16, a fixed learning rate of 1e-05, a dropout probability of 0.3, average cross-entropy loss, and AdamW^35^ optimizer. Since the initial layers of the models only learn very general features, we kept them unchanged and only fine-tuned the last layers for our classification task. We tokenized and fed our input training data to fine-tune the models and then used the fine-tuned models for the test set classification. The model was trained for 5 epochs. Each model was evaluated via 10-fold cross-validation by randomly training using 80% of the data while keeping out 20% for testing to avoid overfitting. Model’s performance was measured in terms precision, recall and F1-score. Since the sample cohort was unbalanced for PSH and FSH across all sites, we used the weighted average from scikit learn python library to compute these measures^36^. All experiments were run in HIPAA-compliant computing environments at the participating sites, equipped with adequate computational resources.

### 2.5 Demographic and diagnostic analysis

We further benchmarked the performance of our NLP and DL tools against a traditional method that relies exclusively on diagnostic codes. At each site we analyzed demographic and diagnosis data on patients in the gold standard corpora. We used the ICD-9/10 diagnosis code (Table S1, Supplementary Materials) for querying STBs in the EHR. This benchmarking process was crucial in assessing the relative effectiveness of the NLP and DL tools in capturing a more comprehensive and accurate picture of patients’ mental health status compared to methods dependent solely on diagnostic codes.

## 3. Results

The patient cohorts are predominantly women across all three study sites: 60.4% at WCM, 63.3% at NM, and 67.7% at UF. The study involved examining 301 notes from 134 patients at WCM, 400 notes from 313 patients at NM, and 400 notes from 341 patients at UF. Notably, demographic variations (Table 1) were evident across the sites, particularly in age, race, and ethnicity. Both WCM (32.8%) and UF (33.4%) had a higher representation of older patients (aged 60 or older) compared to NM (17.9%). The UF cohort (36.4%) had a higher representation of Black patients when compared to WCM (7.5%) and NM (14.1%) cohorts. Additionally, a larger proportion of non-Hispanic patients was observed at UF when compared to WCM and NM sites (96.2% vs 89.6% and 81.5%). Across all sites, more than 90% of the patients had no recorded STB diagnosis (either pre-existing or concurrent with the note date), according to the ICD-9/10 codes in their medical records.

The rule-based NLP tool demonstrated varying performance across different sites (Table 2). Specifically, the macro-average F1-scores ranged from 0.81 to 0.63 for PSH and 0.80 to 0.58 for FSH when evaluated against the corresponding gold standards. Notably, the highest performance for both outcomes was recorded at WCM. The lowest performance for PSH was observed at UF, primarily as a result of a reduced recall of 0.60. Similarly, for FSH, the lowest performance occurred at NM, mainly because of a low recall of 0.44.

The performance of the DL-based tools is shown in Table 3. For PSH, utilizing Bio_ClinicalBERT-based model yielded F1-score of 0.88, 0.73, and 0.88 at WCM, NM and UF, respectively. In contrast, the GatorTron-based model demonstrated superior performance for PSH with F1-score of 0.92, 0.78, and 0.83 at WCM, NM and UF, respectively.

For FSH, both methods showed comparable effectiveness: Bio_ClinicalBERT-based DL achieved F1-score of 0.88, 0.81, and 1.00 at WCM, NM and UF, respectively, while GatorTron-based DL reported slightly higher scores of 0.88, 0.90, 1.00 at WCM, NM and UF, respectively. In the WCM cohort of 134 patients, while manual annotation identified 45 (33.6%) patients with a PSH and 14(10.4%) patients with a FSH, only 1(2.2%) PSH patient and 1(7.1%) FSH patient had relevant ICD codes on or before the notes report date. Conversely, the DL-based GatorTron tool accurately detected 42 (93.3%) of the PSH cases and 12 (85.7%) of the FSH cases from patient notes. No ICD code exists for FSH, precluding direct comparison of underdiagnosis in EHRs. In the NM cohort of 313 patients, while manual annotation identified 97 (31.0%) patients with PSH and 57 (18.2%) with FSH, only 9 (9.3%) PSH and 6 (10.5%) FSH patients had ICD codes for STB on or before the notes report dates. GatorTron, however, successfully identified 78(80.4%) of the PSH cases and 51 (89.5%) of the FSH cases from notes. In the UF cohort of 341 patients, manual annotation identified 64 patients with PSH and 12 with FSH. Of these, only 5 (7.8%) PSH and 0 FSH patients had any ICD code for SI or SB on or before the notes report date. GatorTron, successfully identified 57 (89.0%) of the PSH cases and 12 (100%) of the FSH cases.

## 4. Discussion

Individuals with a PSH or a FSH have an increased risk for future suicide events. However, none of the existing methods reported for the detection of STB specifically looked at the temporal or historical nature of these events. We developed rule-based NLP and DL-based tools to detect PSH and FSH from clinical notes and compared results based on clinical diagnoses. The algorithms were validated by comparison to manually annotated clinical notes of patients with different characteristics from three different academic medical centers in the U.S. For both PSH and FSH, the DL methods showed higher performance than the rule-based NLP method across all three sites. Given that prior efforts to detect STBs from clinical notes did not focus specifically on the historical aspect of suicidality, a direct comparison of the performance of our current tools with existing literature is not feasible. Fernandes et al. developed an NLP method to detect SI and SA using a psychiatric clinical research database and reports a sensitivity of 0.88 and a precision of 0.92 for SI and sensitivity of 0.98 and a precision of 0.83 for SA^22^. Also, using a weak supervision NLP method, efforts from our group recently reported an F1-score of 0.82 for the current SI^21^. Similarly, Carson et al. developed an ML method using terms extracted from clinical notes to identify SB among psychiatrically hospitalized adolescents and reported a sensitivity of 0.83, specificity of 0.22, and AUC of 0.68^18^. While the current rule-based tool demonstrates comparable performance at the development site (WCM), its performance at external sites (NM and UF) was lower, suggesting room for further improvement. The Transformer-based BERT model, on the other hand, has comparable performances at both the development sites and external sites making it a better choice for detecting history of STBs. Moreover, the DL-based tools were fine-tuned on relatively small number of notes (gold standard corpus) from each site which reduces the overall development effort required. In contrast, the rule-based NLP tools relied on an iterative process of manually defining lexicon and implementing specific set of rules using a large development corpus. Error analysis at the three sites further suggests that documentation practices vary widely when reporting temporal aspects of STB. For instance, at NM notes suicidal events documented using a timestamp accounted for the majority of false negative cases and contributed towards the low recall. This includes examples such as “on xx/xx was hospitalized x wk for suicide attempt by overdose”, “Suicidal ideation xx/xx/xxxx”, and “suicide attempt xxxx”.

The DL-tool we developed demonstrated robustness to the varying documentation practices and patient populations presented across the three sites. We analyzed clinical notes from diverse healthcare settings at WCM, NM, and UF to understand documentation practices in different medical environments and clinical settings. WCM’s cohort primarily came from general outpatient settings, focusing on mental health diseases and health maintenance, with detailed notes on personal and family health histories, including mental health and suicidality indicators. At NM, the data encompassed a wider range of settings, including ED, inpatient, and outpatient services. Notes from the ED and inpatient settings often capture more immediate and acute health concerns, potentially including emergent personal mental health crises like STB. ED notes, while comprehensive, varied in family mental health details. Outpatient notes at NM, similar to WCM, would likely include comprehensive health histories of patients but with varying degrees of details regarding mental health, depending on the clinician’s specialty and primary reason for the outpatient visit. At UF, the clinical notes were specifically collected from patients with at least 1 outpatient visit and prescribed opioids in an outpatient setting. In such a setting, provider notes are expected to be centered around oncological care, treatment plans, and follow-ups. However, these notes can also be rich sources of information regarding a patient’s mental health, as dealing with a cancer diagnosis can significantly impact psychological well-being. The likelihood of encountering explicit mentions of personal or family history of STB might be higher in this context, given the profound emotional and psychological implications of cancer on patients and their families. The comparable performance of the DL methods at the three sites, despite the fact that notes were originated at different clinical settings, suggest the general suitability of this method in detecting history of STB from clinical notes.

Previous research indicates that mental health conditions were inadequately recorded as structured ICD or SNOMED codes in EHRs, but were more likely to be documented in patient notes^38^. Our prior study found that among patients identified through NLP-based approach as having PHQ-9 scores—a clinical instrument measuring depression severity—31% showed scores suggesting major depressive disorder, yet lacked a corresponding structured ICD or SNOMED diagnosis code^39^. The current study reveals a similar pattern of underdiagnosis when relying solely on ICD codes.

### Limitations

The study, while advancing the field of suicidality detection using NLP and DL tools through a multi-site approach, faces several limitations. Primarily, the lexicon for STB and history modifiers was restricted, leading to moderate performance at external sites due to missed keywords specific to those locations. This issue is particularly pronounced in self-harm behavior identification, where diverse operational definitions and behaviors like cutting and burning are not uniformly documented. Local customization of the lexicon could enhance algorithm accuracy.

Secondly, the rule-based NLP and DL tools were tested across three academic medical institutions, which may not reflect the broader healthcare system. While the data from NM encompassed diverse clinical settings and specialties, broader testing across various contexts is essential to confirm the efficacy and adaptability of our tools.

Thirdly, modern EHR systems use pre-defined templating component in organizing and documenting clinical notes. The extent to which these templates are included in notes varies widely across the three sites. While notes from WCM and UF are mostly in free-text format with little or no templating components, those from NM exhibit a mix of semi-structured and unstructured formats varying with the type of note. Our rule-based NLP algorithm did not account for possible templating structure and may have impacted negatively on the performance.

## 5. Conclusion

In conclusion, our study highlights the significant potential of rule-based NLP and DL tools in identifying personal and family histories of STB, which are often only recorded in free-text notes within various EHR systems. This approach marks a crucial advancement in suicide prevention efforts. Our findings indicate that traditional methods of structured information collection might miss up to 90% of patients exhibiting suicidal thoughts or behaviors, as these are often only mentioned in unstructured clinical notes. For about 80% of these cases, our developed models prove effective in extracting this critical information, underscoring the value of these tools in enhancing mental health assessments. The DL-based approach achieves a higher and more robust performance across diverse health care systems compared to rule-based NLP.

## Figures and Tables

**Table 1:** Demographics characteristics and ICD diagnosis of patients for which the gold standard corpora were developed and used for evaluating the performance of the NLP and DL methods at the three sites.

	WCM	NM	UF
**Total patients (n)**	134	313	341
**Total notes (N)**	301	400	400
**Avg age (SD) on date of note documented**	48.43(18.26)	44(14.52)	52.18(15.17)
**Age categories**			
< 18	2(1.5%)	1(0.3%)	4(1.2%)
18–39	47(35.1%)	127(40.6%)	66(19.4%)
40–59	41(30.6%)	129(41.2%)	157(46%)
≥60	44(32.8%)	56(17.9%)	114(33.4%)
**Sex**			
Female	81(60.4%)	198(63.3%)	231(67.7%)
Male	53(39.6%)	115(36.7%)	110(32.3%)
**Race**			
White	86(64.2%)	229(73.2%)	203(59.5%)
Black/AA	10(7.5%)	44(14.1%)	124(36.4%)
Native/Alaskan	0(0.0%)	2(0.6%)	NA
Asian	4(3.0%)	5(1.6%)	2(0.6%)
Other/Declined /Unknown	34(25.4%)	32(10.2%)	12(3.5%)
**Ethnicity**			
Hispanic	12(9.0%)	44(14.1%)	10(2.9%)
Non-Hispanic	120(89.6%)	255(81.5%)	328(96.2%)
Other/Declined/Unknow	1(0.7%)	32(10.2%)	3(0.9%)
**Marital Status**			
Single	83(61.9%)	167(53.4%)	NA
Married	30(22.4%)	99(31.6%)	NA
Divorced	13(9.7%)	NA	NA
Widowed	7(5.2%)	NA	NA
Other	1(0.7%)	47(15%)	NA
**ICD diagnosis of SI on analyzed encounter**			
Yes	1(0.7%)	9(2.9%)	5(1.5%)
No	133(99.3%)	304(97.1%)	336(98.5%)
**ICD diagnosis of SB on analyzed encounter**			
Yes	0(0.0%)	6(1.9%)	2(0.6%)
No	134(100.0%)	307(98.1%)	339(99.4%)
**ICD diagnosis of SI before analyzed encounter**			
Yes	1(0.7%)	31(9.9%)	27(7.9%)
No	133(99.3%)	282(90.1%)	314(92.1%)
**ICD diagnosis of SB before analyzed encounter**			
Yes	1(0.7%)	23(7.3%)	17(5.0%)
No	133(99.3%)	290(92.7%)	324(95.0%)
**Provider specialty**			
Psychiatric	31(23.1%)	669(21.1%)	2(0.6%)
Non-Psychiatric	103(76.9%)	247 (78.9%)	339(99.4%)

**Table 2.** Performance of the rule-based NLP tools at the three sites

	WCM	NM	UF
	
**Suicidal History**			
Accuracy	0.82	0.79	0.84
Precision (macro average)	0.81	0.67	0.77
Recall (macro average)	0.83	0.69	0.60
F1-score (macro average)	0.81	0.68	0.63
**Family Suicidal History**			
Accuracy	0.94	0.91	0.98
Precision (macro average)	0.79	0.85	0.99
Recall (macro average)	0.80	0.44	0.62
F1-score (macro average)	0.80	0.58	0.69

**Table 3.** Performance of the Transformer based DL tools at the three sites.

	WCM (n=301)		NM (n=400)		UF (n=400)	
	Bio_ClinicalBERT	GatorTron	Bio_ClinicalBERT	GatorTron	Bio_ClinicalBERT	GatorTron
	
**PSH**						
Accuracy	0.89	0.93	0.74	0.79	0.91	0.87
Precision	0.90	0.93	0.75	0.81	0.86	0.80
Recall	0.89	0.92	0.74	0.79	0.91	0.87
F1-score	0.88	0.92	0.73	0.78	0.88	0.83
**FSH**						
Accuracy	0.90	0.91	0.84	0.91	1.00	1.00
Precision	0.88	0.85	0.79	0.89	1.00	1.00
Recall	0.90	0.91	0.84	0.91	1.00	1.00
F1-score	0.88	0.88	0.81	0.90	1.00	1.00

## Data Availability

The data of this study are not publicly available due to privacy and ethical restrictions. Data to support the findings of this study are available upon reasonable request.
